# Defining the contribution of SNPs identified in asthma GWAS to clinical variables in asthmatic children

**DOI:** 10.1186/1471-2350-14-100

**Published:** 2013-09-25

**Authors:** Asif S Tulah, John W Holloway, Ian Sayers

**Affiliations:** 1 Division of Respiratory Medicine, Queen’s Medical Centre, University of Nottingham, Nottingham NG7 2UH, United Kingdom; 2 Human Genetics and Medical Genomics, Human Development and Health, Faculty of Medicine, University of Southampton, Southampton, UK; 3 Institute of Cellular Medicine, Faculty of Medical Sciences, Newcastle University, Newcastle upon Tyne, UK

**Keywords:** Asthma, ENCODE, eQTL, GWAS, Clinical endpoints

## Abstract

**Background:**

Asthma genome-wide association studies (GWAS) have identified several asthma susceptibility genes with confidence; however the relative contribution of these genetic variants or single nucleotide polymorphisms (SNPs) to clinical endpoints (as opposed to disease diagnosis) remains largely unknown. Thus the aim of this study was to firstly bridge this gap in knowledge and secondly investigate whether these SNPs or those that are in linkage disequilibrium are likely to be functional candidates with respect to regulation of gene expression, using reported data from the ENCODE project.

**Methods:**

Eleven of the key SNPs identified in eight loci from recent asthma GWAS were evaluated for association with asthma and clinical outcomes, including percent predicted FEV_1_, bronchial hyperresponsiveness (BHR) to methacholine, severity defined by British Thoracic Society steps and positive response to skin prick test, using the family based association test additive model in a well characterised UK cohort consisting of 370 families with at least two asthmatic children.

**Results:**

*GSDMB* SNP rs2305480 (Ser311Pro) was associated with asthma diagnosis (p = 8.9×10^-4^), BHR (p = 8.2×10^-4^) and severity (p = 1.5×10^-4^) with supporting evidence from a second *GSDMB* SNP rs11078927 (intronic). SNPs evaluated in *IL33, IL18R1, IL1RL1, SMAD3, IL2RB, PDE4D, CRB1* and *RAD50* did not show association with any phenotype tested when corrected for multiple testing. Analysis using ENCODE data provides further insight into the functional relevance of these SNPs.

**Conclusions:**

Our results provide further support for the role of *GSDMB* SNPs in determining multiple asthma related phenotypes in childhood asthma including associations with lung function and disease severity.

## Background

Asthma is a complex respiratory disease with susceptibility involving the interplay between genetic and environmental factors [1]. The chronic inflammation of the airways is characterised by reversible airflow obstruction and increased hyperresponsiveness of the airways. Genome-wide association studies (GWAS) are currently the preferred method for the identification of genetic determinants in complex multifactorial diseases such as asthma. These studies have the advantage of being able to identify polymorphisms with small effect sizes and localise smaller susceptibility regions due to linkage disequilibrium (LD). The first GWAS for asthma was completed involving 994 childhood-onset asthma patients and 1,243 non-asthma controls and identified single nucleotide polymorphisms (SNPs) within chromosome 17q21 spanning the *ORMDL3/GSDMB* genes [2]. These SNPs showed highly significant association with childhood asthma and were also replicated in two independent cohorts [2]. A study in an Icelandic cohort showed significant association with asthma and a SNP in *IL1RL1*[3] and SNPs in *PDE4D* have also shown association with genome-wide significance with asthma [4]. *IL-13* and *RAD50* on 5q31.1 and the *HLA-DR/DQ* locus at 6p21.3 have been implicated by Li et al. [5]. Other polymorphisms spanning *IL33*, *IL18R1*, *SMAD3*, *IL2RB* and *CRB1* have been proposed by other GWAS using large scale discovery and replication approaches [6,7]. However, due to the phenotype used for case definition, which is often a physician diagnosis or self-report of asthma, there remains a need to determine the contribution of these polymorphisms to clinically relevant endpoints in asthma. This will provide a greater insight into the mechanisms altered in individuals carrying the relevant risk alleles.

One problem that arises from GWAS is that LD patterns can result in multiple polymorphisms at a locus being associated with disease phenotype, even if only one of them is causal. A problem therefore exists in the identification of potential causal variants. This can be simply analysed for polymorphisms within coding regions, as they could potentially affect the polypeptide structure and function and those SNPs in regulatory or intronic regions could affect splice site motifs, transcriptional regulation or RNA stability [8,9]. However, many GWAS have implicated polymorphisms in non transcribed regions far away from annotated genes, where their role is likely to be regulatory [10]. The ENCODE project was established to identify all the functional elements such as protein binding sites, transcripts (coding and non coding) and chromatin marks in the human genome. This represents a valuable resource to provide additional meaning to association data provided from GWAS [11,12]. Taken in consideration with LD data from the 1000 Genomes project [13], computational analysis will enable assignment of more meaningful potential function to SNPs identified from GWAS or those SNPs which are in LD with a GWAS associated SNP [10], to help better prioritise SNPs for functional analysis.

The aim of the present study was to complete a SNP for SNP analysis of the key variants identified in the Caucasian population meeting genome-wide significance and/or replication identified 2007–2011 on clinical variables underlying asthma (Table 1). As these SNPs have previously shown genome-wide association, we also sought to investigate the potential functional significance of these eleven SNPs and those in LD (with these SNPs) by mining the ENCODE dataset [14] and also investigating whether they show association with expression quantitative trait loci (eQTL).

**Table 1:** Key SNPs identified in asthma GWAS included in the current analyses

**Gene SNP**	**Key SNP**	**Non-coded**	**Coded**	**Location**	MAF*	**P-value**	**Phenotype/Reference**	**MAF in UK families**
		**Allele**	**Allele**		**(Control/case)**			
*GSDMB*	rs2305480	C	T	Ser311Pro	0.45	6E-23	Asthma (childhood) [2,6]	0.43
*GSDMB*	rs11078927	C	T	intron	0.45/0.40	7.4E-07	Asthma [2,6]	0.43
*IL33*	rs1342326	T	G	5′ region	0.16	9E-10	Asthma [6]	0.17
*IL33*	rs3939286	G	A	5′ region	0.25/0.28	5.3E-06	Asthma [3]	0.27
*IL18R1*	rs3771166	C	T	intron	0.38	3E-09	Asthma [6]	0.38
*IL1RL1*	rs1420101	G	A	intron	0.37/0.40	5.5E-12	Asthma [3]	0.38
*SMAD3*	rs744910	A	G	intron	0.49	4E-09	Asthma [6]	0.48
*IL2RB*	rs2284033	A	G	intron	0.44	1.1E-08	Asthma [6]	0.43
*PDE4D*	rs1588265	A	G	intron	0.34/0.23	4.3E-07	Asthma [4]	0.29
*CRB1*	rs2786098	C	A	intron	0.15	9.3E-11	Asthma [7]	0.22
*RAD50*	rs2244012	T	C	intron	0.21	3.05E-07	Asthma [5]	0.18

Previous GWAS associations include**;** a chromosome 17 locus (orm1-like protein3 (*ORMDL3*)/gasdermin B (*GSDMB*)), interleukin 33 (*IL33*), a chromosome 2 locus (interleukin 18 receptor (*IL18R*)/interleukin 1 receptor like 1 (*IL1RL1)*), mothers against decaptentaplegic drosophila homolog 3 (*SMAD3*), and IL2 receptor beta (*IL2RB*), phosphodiestase 4D, cAMP specific (*PDE4D*), a chromosome 1 locus (Crumbs homolog 1 precursor (*CRB1)*) and RAD50, S. Cerevisiae, homolog of (*RAD50)*. *MAF* minor allele frequency, *a single MAF is shown where the original paper only reported the MAF in all subjects. The p-value stated is for asthma association in the original study (referenced).

## Methods

### Asthma cohort characteristics

The asthma family cohort was made up of United Kingdom families from the Southampton and Nottingham areas (n = 370 families were used in this study) (Table 2). From the Southampton area, 341 Caucasian families (n = 1508) with at least two biological siblings with physician diagnosed asthma were recruited. This cohort has been described in detail previously [15]. Baseline FEV_1_ (forced expiratory volume in one second) was measured as best of three values within 5% performed using Vitalograph dry-wedge bellows spirometer (Vitalograph Ltd, Buckingham, UK). This was determined 14 days after respiratory tract infection or use of bronchodilator or anti-allergic medication. 46 Caucasian families (n = 184) with at least two biological siblings with physician diagnosed asthma were recruited from the Nottingham area [15]. FEV_1_ was defined as the best of three values. All subjects were classified according to British Thoracic Society Step Guidelines (BTS steps, ranging from step 1 to step 5) based on physician prescribed medication [16]. Ethical approval for the Southampton subjects was obtained from the Southampton and South West Hampshire and the Portsmouth and South East Hampshire Local Research Ethics Committees and for the Nottingham subjects, the Nottingham University Medical School Ethics Committee. Written informed consent for study participation was obtained from participants (from the parent/guardian for the children).

**Table 2:** Clinical characteristics of UK families

	**Pedigrees**	**Sibling 1**	**Sibling 2**
Age (mean years), ±SD	26.2 ± 1.6	13.3 ± 4.4	10.3 ± 4.6
Gender (% female)	50.8	54.6	53.7
Asthma (%, doctor diagnosed)	61.6	100	100
FEV_1_ (% predicted, ±SD)	ND	95.4 ± 15.6	96.2 ± 14.9
Positive skin prick test (%)	62.5	73.9	65.1
Log total serum IgE (mean ± SD)	2.04 ± 0.7	2.33 ± 0.7	2.33 ± 0.7
Eczema (%, questionnaire)	41.1	53.5	55.1
Hay fever (%, questionnaire)	50.2	64.9	47.4
BTS (%) step 1	19.1	24.9	23.8
• Step 2	32.5	52.7	55.7
• Step 3	6.1	11.6	10.2
• Step 4	1.8	4.6	1.4
• Step 5	2.9	4.6	5.3
PC_20_ (4 mg/ml,%)		46.9	59.9
n	1578	370	361

The recruitment and clinical characterisation of these subjects has been extensively described elsewhere [15]. PC_20_ (4 mg/ml, %) only available in Southampton families.

### SNP selection and genotyping

Eleven SNPs were chosen that have been identified from asthma GWAS identified 2007–2011 (Table 1). These were in eight loci; *ORMDL3*/*GSDMB*, *IL33*, *IL18R*/*IL1RL1*, *SMAD3*, *IL2RB*, *PDE4D*, *CRB1* and *RAD50*. SNPs were genotyped using KASPar technology by KBiosciences (Hertfordshire, UK). For quality control of genotyping data, Chi square was used to test for any deviation between the observed genotype frequencies and the expected, under the Hardy Weinberg Equilibrium.

### Association analyses

Association analysis in the family cohorts between the GWAS SNPs and asthma-related traits was conducted using the family based association test (FBAT) software (version 1.5.1) [17] in the additive model. Clinically relevant endpoints including; asthma diagnosis, Forced Expiratory Volume in one second (FEV_1_% Predicted), bronchial hyperresponsiveness to methacholine (BHR), British Thoracic Society (BTS) defined severity [18] and atopy (positive response to skin prick test to any one of a panel of allergens as outlined [15]). We completed power calculations based on the method of Risch & Merikangas [19]. There was between 0.93 and 0.98 power to detect an association with asthma with a significance level of p = 0.05 and a relative risk of 1.5. There was between 0.51 and 0.69 power to detect an association with p = 0.05 and a relative risk of 1.25 (calculations based on minor allele frequency (min 0.17 to max 0.48 observed). Power curves for these MAF are provided in Additional file 1. To address multiple testing, we used Bonferonni correction, which considered p < 9.1×10^-4^ significant. This was based on our eleven genotyped SNPs analysed for five phenotypes (0.05/55 total tests = 9.1×10^-4^ as a threshold p-value).

### Bioinformatic analysis of genotyped SNPs

HaploReg [14] (a web based tool) was used to provide information about the sequence surrounding a SNP. It has been developed to mine chromatin state information from nine human cell lines; GM12878, H1, HMEC, HSMM, HepG2, Huvec, K562, NHEK and NHLF. The tool provides information on SNPs that are in LD with the queried SNP (r^2^ was set to 1 for these analyses and the population chosen for analyses was of European descent) and also gives information on which of these SNPs lie in enhancer histone marks, DNase sites and protein binding regions. It can also inform on the conservation of the relevant sequence and any changes to regulatory motifs based on the SNP allele changes. Each of the eleven genotyped SNPs were entered into HaploReg and analysis was completed with r^2^ = 1. A more detailed analysis was completed for any SNP which showed significant association in the population study.

We used the program mRNA by SNP browser (version 1.0.1) to investigate whether any of the GWAS SNPs genotyped as part of this study were associated with regions of known asthma expression quantitative trait loci (eQTL). This program incorporates a database of eQTL from asthma studies and provides a graphical output to browse data from GWAS. It provides association data between 54,675 transcripts and 406,912 SNPs. Statistics are provided for those SNPs with association p < 0.001. We used the program to search for each SNP in turn by inputting either the gene name or the SNP rs number, looking to see whether the association reached genome-wide significance (p < 10^-8^) [20]. We also used the eQTL browser from the University of Chicago (http://eqtl.uchicago.edu/cgi-bin/gbrowse/eqtl/) which considers eQTLs from different tissue sources. A second stage of this analysis was to investigate whether these same SNPs showed genome-wide association with eQTL from a genome-wide search in 1,111 human lung samples [21].

## Results

### Clinical characteristics and genotyping

Clinical characteristics for the asthma families are shown in Table 2. Mean age was 13.3 years and 10.3 years old, mean FEV_1_ (% Predicted) was 95.4 ± 15.6 and 96.2 ± 14.9 and % atopy (SPT) was 73.9% and 65.1% for sibling 1 and 2 respectively. These asthmatic children had predominantly mild-moderate disease as outlined by BTS guidelines. Genotyping was completed using KASPar (KBioscience) and quality control completed as outlined in previous studies [15]. All SNPs were in Hardy Weinberg equilibrium and allele frequencies in the UK families were similar to those previously reported (Table 1).

### Association with asthma, FEV_1_ and BHR

A *GSDMB* SNP at the 17q21 locus, rs2305480 (Ser311Pro) was associated with asthma diagnosis, with the T allele conferring risk to develop asthma as previously reported [6] (p = 8.9×10^-4^, *z* = -3.23) (Table 3). Another *GSDMB* SNP rs11078927 (intronic) showed similar findings with asthma but did not reach statistical significance (p = 9.5×10^-4^, *z* = -3.23) although this SNP was significantly associated with FEV_1_ (% Predicted) (p = 8.7×10^-4^, *z* = -3.33). Significant association was also observed with rs2305480 (*GSDMB*, Ser311Pro) and BHR (PC_20_ methacholine, 4 mg/ml) (p = 8.2×10^-4^, *z* = -3.35). No other SNP association survived correction for multiple testing (Table 3).

**Table 3:** **Association of key GWAS SNPs with asthma diagnosis, percent predicted FEV**_**1 **_**and bronchial hyperresponsiveness to methacholine**

**SNP**	**Gene**	**Asthma**	**Z**	**P**	**FEV**_**1**_	**Z**	**P**	**BHR**	**Z**	**P**
		**Fam**	**Score**	**value**	**Fam**	**Score**	**value**	**Fam**	**Score**	**value**
rs2305480 C/T	*GSDMB*	265	-3.323	**8.9×10**^**-4**^	274	-3.247	1.1×10^-3^	240	-3.345	**8.2×10**^**-4**^
rs11078927 C/T	*GSDMB*	272	-3.305	9.5×10^-4^	278	-3.330	**8.7×10**^**-4**^	244	-3.238	1.2×10^-3^
rs1342326 T/G	*IL33*	176	+1.114	0.265	180	+1.266	0.205	156	+1.412	0.158
rs3939286 G/A	*IL33*	198	+0.412	0.680	204	+0.544	0.586	176	+0.693	0.489
rs3771166 C/T	*IL18R1*	263	-1.475	0.140	270	-1.637	0.102	228	-1.416	0.157
rs1420101 G/A	*IL1RL1*	261	+0.899	0.369	264	+0.658	0.511	223	+0.737	0.461
rs744910 A/G	*SMAD3*	264	+0.116	0.907	271	+0.823	0.411	234	-0.166	0.868
rs2284033 A/G	*IL2RB*	262	+0.825	0.409	267	+0.416	0.677	233	+0.778	0.436
rs1588265 A/G	*PDE4D*	233	-0.380	0.703	239	-0.056	0.956	205	-0.506	0.613
rs2786098 C/A	*CRB1*	202	-1.702	0.089	204	-1.324	0.185	178	-2.377	0.017
rs2244012 A/G	*RAD50*	187	+0.953	0.340	189	+0.659	0.510	164	+1.075	0.282

FBAT was used to test for association using the additive model [17]. *SNP* single nucleotide polymorphism, *MAF* minor allele frequency, *Fam* number of families in analysis (≥10 families). Asthma = Doctor diagnosed asthma, FEV_1_ = FEV_1_ percent predicted, BHR = PC_20_ (methacholine 4 mg/ml) only available in Southampton Families, Bonferroni correction, P < 9.1×10^-4^ considered significant (bold).

### Association with asthma severity and atopy

Both *GSDMB* SNPs showed significant association with severity of disease defined by BTS steps (1–5), both with the same direction of association; rs2305480 (p = 1.5×10^-4^, *z* = 3.79) and rs11078927 (p = 2.2×10^-4^, *z* = -3.70). No other SNP association survived correction for multiple testing (Table 4).

**Table 4:** Association of key GWAS SNPs with asthma severity defined by BTS steps and atopy

**SNP**	**Gene**	**Severity**	**Z**	**P**	**SPT**	**Z**	**P**
		**Fam**	**Score**	**value**	**Fam**	**Score**	**value**
rs2305480 C/T	*GSDMB*	272	-3.791	**1.5×10**^**-4**^	220	-3.164	1.6×10^-3^
rs11078927 C/T	*GSDMB*	277	-3.701	**2.2 ×10**^**-4**^	227	-3.133	1.7×10^-3^
rs1342326 T/G	*IL33*	180	+1.378	0.168	144	+2.585	9.7×10^-3^
rs3939286 G/A	*IL33*	204	+0.347	0.729	164	+2.146	0.032
rs3771166 C/T	*IL18R1*	269	-2.021	0.043	219	-1.683	0.092
rs1420101 G/A	*IL1RL1*	263	+1.577	0.115	218	+0.449	0.653
rs744910 A/G	*SMAD3*	268	+0.386	0.699	215	+0.157	0.875
rs2284033 A/G	*IL2RB*	268	+1.233	0.218	216	+1.265	0.206
rs1588265 A/G	*PDE4D*	235	-0.712	0.477	195	-0.360	0.719
rs2786098 C/A	*CRB1*	202	-1.021	0.307	158	-1.886	0.059
rs2244012 A/G	*RAD50*	188	+0.941	0.347	159	+1.242	0.214

FBAT was used to test for association using the additive model [17]. *SNP* single nucleotide polymorphism, *MAF* minor allele frequency, *Fam* number of families in analysis (≥10 families). Severity = BTS classification (1–5) [18], SPT = positive skin prick test to one or more allergen. Bonferroni correction, P < 9.1×10^-4^ considered significant (bold).

### Bioinformatic analysis of SNPs using data from ENCODE

We sought to investigate whether data collected from the ENCODE Consortium [12] could enrich the potential functional significance of our genotyped GWAS associated SNPs and also those SNPs which were in LD (Table 5). The two *GSDMB* SNPs were found to be in the same LD block (r^2^ = 1), with 24 other SNPs. rs2244012 (*RAD50*/intronic) was found to be in an LD block with 50 other SNPs. The other genotyped SNPs were in smaller LD blocks. Most of our genotyped SNPs analysed were found to have at least one SNP (in their LD block) existing in an enhancer histone mark site, DNase site or a region where protein binding consensus sequences exist, highlighting a potential functional effect of these particular SNPs. There was evidence for LD SNPs altering regulatory motifs, with 14 SNPs in the *GSDMB* LD block and 25 SNPs in the *RAD50* LD block the highest numbers (Table 5).

**Table 5:** Analysis of key GWAS SNPs with data from ENCODE

**SNP**	**Gene locus/location**	**Number of SNPs in LD (r**^**2**^ **= 1) (no.)**	**GERP Conservation**	**Enhancer histone marks (no. of SNPs in regions)**	**DNase (no. of SNPs in regions)**	**Proteins bound (no. of SNPs in regions)**	**Motifs changed (no. of SNPs with motif changed regions)**
			**(no. of SNPs conserved)**				
rs2305480 C/T	*GSDMB*/Ser311Pro	26	2	3	1	0	14
rs11078927 C/T	*GSDMB*/intronic	26	2	3	1	0	14
rs1342326 T/G	*IL33*/5′-region	7	0	1	2	2	4
rs3939286 G/A	*IL33*/5′-region	1	0	0	0	0	1
rs3771166 C/T	*IL18R1*/intronic	16	0	2	1	0	9
rs1420101 G/A	*IL1RL1*/intronic	6	1	2	1	1	1
rs744910 A/G	*SMAD3*/intronic	3	0	1	2	2	1
rs2284033 A/G	*IL2RB*/intronic	3	0	0	3	0	1
rs1588265 A/G	*PDE4D*/intronic	14	2	0	1	0	5
rs2786098 C/A	*CRB1*/intronic	11	0	0	3	0	7
rs2244012 A/G	*RAD50*/intronic	51	2	6	9	5	25

The 11 SNPs genotyped as part of this study were entered into the HaploReg tool to investigate whether SNPs in the same LD block were likely to affect chromatin structure and regulatory element binding motifs.

### Analyses of asthma associated *GSDMB* SNPs using ENCODE data

As the two *GSDMB* SNPs (rs2305480/Ser311Pro and rs11078927/intronic) showed significant association in our study, we sought to further investigate potential functional implications using the ENCODE dataset. These two *GSDMB* SNPs themselves were not predicted to be important from a regulatory role, however they were in a LD block with 24 other SNPs. Almost all of the SNPs in this LD block were in non-coding/intronic regions, with the exception of rs10852935, which is a synonymous polymorphism in *ZPBP2* and the genotyped rs2305480/Ser311Pro (Table 6). Twenty four of these SNPs were not conserved (according to the GERP conservation score). Three SNPs were located in enhancer histone marks (rs12709365 and rs13380815 both *ZPBP2*/intronic and identified in cell line GM12878), rs4795399 (*GSDMB*/intronic) was identified in a HepG2 cell line and also located in a DNase site identified in a human hepatocyte cell line (Huh7.5) and a human leukaemic cell line (CMK). While no SNPs were predicted to occur in a protein binding regions, 14 SNPs were predicted to alter regulatory binding motifs (Table 6).

**Table 6:** **Analysis of the *GSDMB *LD block (r**^**2 **^**=1) using data from ENCODE**

**SNP**	**Gene locus/location**	**Chromosomal location (hg19)**	**GERP conservation (Y/N)**	**Enhancer histone marks**	**DNase**	**Proteins bound**	**Motifs changed**
rs12709365 A/G	*ZPBP2*/intronic	17:38027400	N	GM12878	N	N	N
rs13380815 A/G	*ZPBP2*/intronic	17:38027583	N	GM12878	N	N	N
rs11870965 T/A	*ZPBP2*/intronic	17:38030205	N	N	N	N	Myb
rs10852935 C/T	*ZPBP2*/synonymous	17:38031674	Y	N	N	N	N
rs10852936 C/T	*ZPBP2*/intronic	17:38031714	N	N	N	N	Foxp1, Foxq1
rs36095411 T/G	*ZPBP2*/intronic	17:38031865	N	N	N	N	9 motifs altered
rs34189114 C/T	*ZPBP2*/intronic	17:38032460	N	N	N	N	N
rs35736272 T/C	*ZPBP2*/intronic	17:38032680	Y	N	N	N	N
rs9907088 G/A	*ZPBP2*/downstream	17:38035116	N	N	N	N	Sox
rs36038753 G/T	*ZPBP2*/downstream	17:38035370	N	N	N	N	Dobox4
rs35569035 C/T	*ZPBP2*/downstream	17:38036524	N	N	N	N	Pax4
rs9904624 A/G	*ZPBP2*/downstream	17:38036586	N	N	N	N	N
rs12232497 T/C	Intergenic	17:38040119	N	N	N	N	N
rs12232498 T/C	Intergenic	17:38040363	N	N	N	N	Lhx3
rs12941333 C/T	Intergenic	17:38040534	N	N	N	N	Pou3f3, Pou2f1
rs2872507 G/A	Intergenic	17:38040763	N	N	N	N	N
rs12936409 C/T	Intergenic	17:38043649	N	N	N	N	N
rs8069176 G/A	*GSDMB*/downstream	17:38057197	N	N	N	N	Gfi1, Mafk, Pax4
rs4795399 T/C	*GSDMB*/intronic	17:38061439	N	HepG2	Huh7.5,CMK	N	Pxx6, Pax
**rs2305480 G/A**	***GSDMB*/Ser311Pro**	**17:38062196**	**N**	**N**	**N**	**N**	**N**
rs62067034 C/T	*GSDMB*/intronic	17:38063738	N	N	N	N	SREBP, LXR
**rs11078927 C/T**	***GSDMB*/intronic**	**17:38064405**	**N**	**N**	**N**	**N**	**N**
rs11078928 T/C	*GSDMB*/intronic	17:38064469	N	N	N	N	Foxp1
rs4795400 C/T	*GSDMB*/intronic	17:38067020	N	N	N	N	N
rs77749396 G/C	*GSDMB*/intronic	17:38073840	N	N	N	N	ZNF219
rs9303279 G/C	*GSDMB*/intronic	17:38073968	N	N	N	N	Obox3

The two GSDMB SNPs genotyped as part of this study (rs2305480 and rs110788927) were part of a wider LD block containing 26 SNPs in total spanning 46,568 bp. These SNPs were analysed using HaploReg to see if they atlered any binding sites for regulatory elements or other proteins and also whether they affected chromatin states and their conservation according to GERP. The CEU frequency from 1000 Genomes Project =0.48 for all SNPs. CMK: human leukaemic cell line; *GERP* genomic evolutionary rate profiling score; GM12878: lymphoblastoid cell line; *GSDMB* gasdermin-domain containing protein family B; HepG2: human hepatocellular liver carcinoma cell line; Huh7.5: human hepatocellular cell line; *ZPBP2* zona pellucid binding protein 2 gene. SNPs genotyped in the current study are shown in bold.

### Analysis of asthma eQTL data

We searched for the eleven SNPs genotyped as part of this study in turn using the mRNA by SNP browser resource, looking to see whether the SNP was associated with the effect meeting genome-wide significance (p < 10^-8^). Information for three of the SNPs (rs230548/*GSDMB*, rs2886098/*CRB1* and rs2244012/*RAD50*) was not covered by the database and there was no information for SNPs in LD with these. Another two SNPs were also not covered by the database, however there was information for a SNP in high LD. For rs11078927/*GSDMB*, rs10445308 was in LD (r^2^ = 1.0) and this SNP showed genome-wide significance with multiple ORMDL3 probes (all C-allele); 223259_at (p = 4.4×10^-13^, LOD = 11.39, H^2^ = 14.75) and probe 235136_at (p = 8.0×10^-12^, LOD = 10.16, H^2^ = 13.3) with average ORMDL3 (p = 3.9×10^-22^, LOD = 20.32, H^2^ = 26.36). For rs3771166/*IL18R1*, rs54988956 was in LD (r^2^ = 0.96), but this was not associated with genome-wide significance with any probe. For the other six SNPs, there was no genome-wide significance. Using the University of Chicago eQTL resource, data was available for the two *GSDMB* SNPs (rs2305480 and rs11078927) which provided some evidence that these were eQTLs and exon-QTLs in lymphoblastoid cell lines, monocytes, liver, brain and T-cells. More specifically, both rs2305480 and rs11078927 were eQTL for *IKZF3*, *ORMDL3* and an exon-QTL for *KRT222P*, *NR1D1* and *ORMDL3*. Only three of our eleven genotyped SNPs (rs2305480/*GSDMB*, rs3939286/*IL33* and rs744910/*SMAD3*) were included in the genotyping platform for the genome-wide search for eQTL in 1,111 human lung samples [21]. None of these SNPs were found in the reported data meeting 10% False Discovery Rate (FDR). As a further analysis we also searched this resource for any of the SNPs in high LD (r^2^ = 1) with our genotyped SNPs. rs1337167/*CRB1* met the 10% FDR. This intron SNP was predicted to alter an AP-1 transcription factor binding site according to HaploReg.

## Discussion

GWAS have been successful in identifying susceptibility genes for asthma through the use of large scale population cohorts and replication approaches [2-7]. However, there remains a need to determine the contribution of these polymorphisms to clinically relevant endpoints in asthma as opposed to disease diagnosis. We adopted a two step process where we selected SNPs from key genes in the Caucasian population which have met genome-wide significance and/or with replication, namely eleven SNPs in the following genes; *ORMDL3*/*GSDMB*, *IL33*, *IL18R*/*IL1RL1*, *SMAD3*, *IL2RB*, *PDE4D*, *CRB1* and *RAD50.* These SNPs were genotyped in a UK asthma family cohort with at least two affected siblings, to test for association with clinically relevant endpoints in asthma. Our data provide support for the original *GSDMB* association; however SNPs in the other genes did not show association after correction for multiple testing. Secondly, as these SNPs have previously shown association at a genome-wide level, we also investigated the potential for these SNPs and those in LD to affect chromatin states and alter regulatory motifs/binding sites to try to assign potential function to these associations. This analysis used recently reported data from the ENCODE project. These data support the hypothesis that SNPs associated in GWAS may be tagging SNPs for the actual causative variant, but also provide tentative evidence that these genotyped SNPs may themselves play a functional role e.g. *GSDMB* rs2305480 (Ser311Pro).

GWAS have the advantage over linkage studies in that they can identify polymorphisms with small effect sizes and localise smaller susceptibility loci as LD only generally spans <500 kb [22]. Since the identification of childhood asthma associated variants on chromosome 17q21 [2], many other GWAS have been completed in different populations implicating variants spanning different genes (see above). One problem with these studies lies in defining the contribution of these associated variants to clinical endpoints in asthma. To this end we have completed a SNP for SNP analysis of key variants identified in the Caucasian population meeting genome-wide significance or where there was replication, focussing on GWAS from 2007–2011 (Table 1). We utilised a well characterised UK family based asthma cohort [15] to test for association with asthma diagnosis and the following clinical endpoints: FEV_1_ (% predicted), BHR (to methacholine), BTS defined severity and positive SPT to one or more allergens, see [15]. These data show significant association for the two *GSDMB* SNPs; rs2305480 (Ser311Pro) and rs11078927 (intron) across more than one phenotype, with other phenotypes showing association which does not reach significance after correction for multiple testing. These data both replicate and extend previous findings [2,6] and identify this chromosome 17 locus as containing genetic determinants underlying multiple clinical features of asthma, including FEV_1_ (% Predicted) and disease severity defined by BTS. These two polymorphisms are in high LD suggesting these associations represent a single locus. The BHR association (rs2305480) is supported by different SNPs in this gene also showing association with this phenotype, albeit in different ethnic populations [23,24]. Interestingly, a recent study has found association between rs2305480 and wheezing phenotypes in asthmatic children, but did not find association with intermediate phenotypes such as atopy or lung function [25].

When interpreting the results of GWAS, the associated variant could be tagging another variant in a larger region of LD [26]. This is particularly important as it is common that only a few variants in a haplotype block are present on genotyping platforms. Methods such as imputation and using genotyping data from the 1000 Genomes project can help to further characterise these patterns [27]. However, once these variants are identified it is still necessary to assign potential function and with many associated variants from GWAS residing in non-transcribed regions, they are likely to play a regulatory role in gene expression. We used HaploReg [14] to screen chromatin data, conservation data and regulatory motif databases which was obtained from the ENCODE project to see if any biological plausible role could be assigned to the SNPs genotyped in this study and those SNPs which were in LD with r^2^ = 1. Our results show that these SNPs in LD can potentially lead to changes in regulatory element binding motifs affecting the transcription of genes positively or negatively and there is also the suggestion that alterations in enhancer histone marks or DNase sites could be affected. The non-synonymous SNP (Ser 311 Pro) is predicted to be deleterious to protein structure (Polyphen) in Gasdermin B suggesting a potential functional mechanism altering protein function. This may be occurring at the mRNA level (altering the efficiency of splicing) as this polymorphism is predicted to be located in an exonic splicing enchancer site (FuncPred). Both *GSDMB* SNPs are in high LD with several SNPs spanning *ZPBPZ* and *GSDMB* which result in alterations in enhancer and transcription factor binding sites. However the rs2305480 and rs11078927 SNPs themselves are not predicted to have any regulatory role (ENCODE data). Gasdermin-family proteins have been implicated in TGFβ1 signalling and epithelial cell apoptosis [28], with increased airway cell apoptosis reported in severe asthma [29]. The extended chromosome 17 locus has also been implicated previously as containing SNPs associated with asthma spanning three main genes*; ZPBP2/GSDMB/ORMDL3* and it is still unclear which genes/polymorphisms underlie these associations. The finding that the *GSDMB* SNPs including a non-synonymous SNP are particularly relevant to clinical endpoints including lung function, BHR and severity in our childhood asthma cohort is interesting as few recent studies have genotyped this GWAS SNP or investigated clinical outcomes with the same criteria such as BTS used in this study [24,30]. However, a recent study has investigated the effect of rs7216389 (which is in linkage disequilibrium with both *GSDMB* SNPs in this study) with respect to severe asthma subjects classified with poorly controlled disease whilst taking high doses of inhaled corticosteroids, long-acting bronchodilators and short acting β_2_ agonists and showed association [31]. Similarly, while not meeting genome-wide association significance, we have previously provided evidence that rs2305480 is associated with severe asthma (p = 5.5×10^-5^, [32]), although we do acknowledge that the criteria for BTS severity is defined in part by lung function and so this association with severe asthma may be related to a synergistic effect. These data support previous studies that identified the chromosome 17 locus as containing markers of childhood onset disease [2,6,24,30], BHR [24] and elevated total serum IgE [24] in diverse ethnic populations.

Interestingly, no other SNP survived correction for multiple testing for any phenotype analysed, including asthma diagnosis and there was no indication of any other SNP showing a trend towards significance. *CRB1* rs2786098 (p = 0.089 in the current study) has shown replicated association in multiple childhood cohorts, but did not reach statistical significance in our study. Perhaps this lack of association may be due to power (MAF 0.22, see earlier). One potential explanation for the lack of association for asthma and related phenotypes for the majority of SNPs is that many of the SNPs/genes were identified in adult asthma populations and the relative contribution to childhood asthma remains unclear at this time. However, in the results not meeting conventional correction for multiple testing, *IL33* SNP rs1342326G (25.7 kb from gene transcription start site (TSS)) showed a level of association with atopic asthma defined by positive skin prick test and clinical diagnosis (p = 0.0097, Table 4). These data are in line with the accumulating evidence suggesting a role of IL33 in allergic mechanisms in asthma (Reviewed in [33]) although we cannot exclude that this is a false positive. Similarly, while not surviving correction, suggestive evidence for SNP associations for *CRB1*/BHR, *IL18R*/severity and *IL33*/SPT were apparent (p < 0.05), which may represent true associations.

Studies have shown the potential for GWAS SNPs to be tagging SNPs associated with eQTL [10]. Our analyses did provide some support for this particularly for the two *GSDMB* SNPs (rs2305480 and rs11078927) when eQTL analyses was completed across multiple cell types and tissues. These eQTL analyses identified regulation of multiple genes in the region, demonstrating the need for further work to define the biology underlying these genetic signals.

It is important to note the limitations of our study, while a SNP for SNP approach focussed to the pivotal SNPs from the GWAS has strengths to directly replicate and extend previous findings we acknowledge the assumption that the linkage disequilibrium pattern between the discovery Caucasian population and our UK population will be the same, which may at least in part explain the inability to detect associations with all variants. Similarly, we did not genotype all GWA significant SNPs identified in each of the regions. We acknowledge the reduced power of the current study using 370 families is a limitation potentially explaining while several SNPs showed nominal significance and did not meet stringent statistical criteria. A cohort of 602 families would be required to provide a power of 0.99 for the lowest MAF observed in the current study (relative risk of 1.5 and significance level of 0.05). We also acknowledge that the design of this study compared to discovery cohorts using case/control design also has reduced power, however we consider the use of an alternative association design i.e. family based to those previously published a strength. Furthermore the lack of assessing gene-environment interactions in GWAS and the current study may also account for not replicating previous observed effects due to differential environmental exposures between study populations. Finally, the potential for winners curse bias is also possible for direct replication of e.g. association with asthma diagnosis.

## Conclusions

In summary, this study has provided a greater insight into the relative contribution of asthma GWAS SNPs to clinically relevant endpoints in asthmatic children and further defines the diverse role of *GSDMB* SNPs particularly in lung function and disease severity.

## Abbreviations

BHR: Bronchial hyperresponsiveness; BTS: British Thoracic society; CRB1: Crumbs homolog 1 precursor; DENND1B: Denn/madd domain-containing 1B; ENCODE: ENcyclopedia Of DNA elements; eQTL: Expression quantitative trait loci; FBAT: Family based association test; FEV1 (% Predicted): forced expiratory volume in one second (percent predicted); GSDMB: Gasdermin B; GWA: Genome-wide association; GWAS: Genome-wide association study; IgE: Immunoglobulin E; IL18R: Interleukin 18 receptor; IL1RL1: Interleukin 1 receptor like 1; IL2RB IL2: Receptor beta; IL33: Interleukin 33; LD: Linkage disequilibrium; MAF: Minor allele frequency; ORMDL3: Orm1-like protein 3; PDE4D: Phosphodiestase 4D, cAMP specific; Pro: Proline (amino acid); RAD50 S. Cerevisiae: homolog of (*RAD50)*; SPT: Skin prick test; SMAD3: Mothers against decaptentaplegic drosophila homolog 3; SNP: Single nucleotide polymorphism; Ser: Serine (amino acid); TGFβ1: Transforming growth factor beta 1; TSS: Transcription start site; ZPBP2: Zona pellucid binding protein 2 gene.

## Competing interests

The authors declare that they have no competing interests.

## Authors’ contributions

IS, JWH and AST designed the study and drafted the manuscript. AST completed the analyses. All authors contributed to the final version of the manuscript. All authors read and approved the final manuscript.

## Pre-publication history

The pre-publication history for this paper can be accessed here:

http://www.biomedcentral.com/1471-2350/14/100/prepub

## Supplementary Material

Additional file 1 **Power of the study based on different relative risks for minor allele frequencies of 0.17 and 0.48.** Cohort size n = 370 families and p-value of 0.05. Calculation based on the method of Risch & Merikangas. Click here for file
