# Supplementary material for: Defining the contribution of SNPs identified in asthma GWAS to clinical variables in asthmatic children
Source: BMC Med Genet. 2013 Sep 25;14:100. doi: 10.1186/1471-2350-14-100 (PMC3849932; doi:10.1186/1471-2350-14-100)
Supplement: Additional file 1 — Power of the study based on different relative risks for minor allele frequencies of 0.17 and 0.48. Cohort size n = 370 families and p-value of 0.05. Calculation based on the method of Risch & Merikangas. [file 1471-2350-14-100-S1.doc]

**Additional Files**

**Defining the contribution of SNPs identified in asthma GWAS to clinical variables in asthmatic children**

Asif S. Tulah^1,3^, John W. Holloway^2^, and Ian Sayers^1§^

*^1^Division of Respiratory Medicine, University of Nottingham, Queen’s Medical Centre, Nottingham, United Kingdom*

*^2^Human Genetics and Medical Genomics, Human Development and Health, Faculty of Medicine, University of Southampton, Southampton, United Kingdom*

*^3^Institute of Cellular Medicine, Faculty of Medical Sciences, Newcastle University, Newcastle upon Tyne, United Kingdom*

^§^**Corresponding author:**

Dr. Ian Sayers

Division of Respiratory Medicine,

Queen’s Medical Centre, University of Nottingham,

Nottingham, NG7 2UH, UK,

External tel: 0115 82 31066, Fax: 0115 82 31059

E-mail: ian.sayers@nottingham.ac.uk

**Additional files**

**Additional File 1 –** Power of the study based on different relative risks for minor allele frequencies of 0.17 and 0.48. Cohort size n=370 families and p-value of 0.05. Calculation based on the method of Risch & Merikangas .

1. Risch N, Merikangas K: **The future of genetic studies of complex human diseases**. *Science* 1996, **273**(5281):1516-1517.
